# PEA‐15 (Phosphoprotein Enriched in Astrocytes 15) Is a Protective Mediator in the Vasculature and Is Regulated During Neointimal Hyperplasia

**DOI:** 10.1161/JAHA.117.006936

**Published:** 2017-09-11

**Authors:** Fiona H. Greig, Simon Kennedy, George Gibson, Joe W. Ramos, Graeme F. Nixon

**Affiliations:** ^1^ School of Medicine Medical Sciences and Nutrition University of Aberdeen United Kingdom; ^2^ Institute of Cardiovascular and Medical Sciences University of Glasgow United Kingdom; ^3^ Department of Cardiothoracic Surgery Aberdeen Royal Hospital Aberdeen United Kingdom; ^4^ Cancer Biology Program University of Hawaii Cancer Centre University of Hawaii at Mānoa Honolulu HI

**Keywords:** mitogen‐activated protein kinase pathway, neointimal hyperplasia, proliferation, vascular smooth muscle, Cell Signalling/Signal Transduction, Smooth Muscle Proliferation and Differentiation, Restenosis

## Abstract

**Background:**

Neointimal hyperplasia following angioplasty occurs via vascular smooth muscle cell proliferation. The mechanisms involved are not fully understood but include mitogen‐activated protein kinases ERK1/2 (extracellular signal–regulated kinases 1 and 2). We recently identified the intracellular mediator PEA‐15 (phosphoprotein enriched in astrocytes 15) in vascular smooth muscle cells as a regulator of ERK1/2‐dependent proliferation in vitro. PEA‐15 acts as a cytoplasmic anchor for ERK1/2, preventing nuclear localization and thereby reducing ERK1/2‐dependent gene expression. The aim of the current study was to examine the role of PEA‐15 in neointimal hyperplasia in vivo.

**Method and Results:**

Mice deficient in PEA‐15 or wild‐type mice were subjected to wire injury of the carotid artery. In uninjured arteries from PEA‐15–deficient mice, ERK1/2 had increased nuclear translocation and increased basal ERK1/2‐dependent transcription. Following wire injury, arteries from PEA‐15–deficient mice developed neointimal hyperplasia at an increased rate compared with wild‐type mice. This occurred in parallel with an increase in a proliferative marker and vascular smooth muscle cell proliferation. In wild‐type mice, PEA‐15 expression was decreased in vascular smooth muscle cells at an early stage before any increase in intima:media ratio. This regulation of PEA‐15 expression following injury was also observed in an ex vivo human model of hyperplasia.

**Conclusions:**

These results indicate, for the first time, a novel protective role for PEA‐15 against inappropriate vascular proliferation. PEA‐15 expression may also be repressed during vascular injury, suggesting that maintenance of PEA‐15 expression is a novel therapeutic target in vascular disease.

**Clinical Perspective**

**What Is New?**

- The intracellular mediator PEA‐15 (phosphoprotein enriched in astrocytes 15) is an important regulatory protein in arteries and acts as a protective mechanism against the development of restenosis.
- PEA‐15 expression is decreased during neointimal development, leading to increased proliferation of vascular smooth muscle cells.

**What Are the Clinical Implications?**

- Maintaining PEA‐15 expression (by mechanisms yet to be determined) following arterial stent placement could decrease the development of neointimal hyperplasia by preventing excessive vascular smooth muscle cell proliferation.

Arterial occlusion caused by atherosclerotic plaque formation is typically treated using balloon angioplasty and arterial stent placement. Approximately 25% to 30% of patients subsequently require reintervention to treat restenotic arteries.^1^ Restenosis is predominantly the result of excessive vascular smooth muscle cell (VSMC) proliferation, initiated in part by an inflammatory component, that leads to neointimal hyperplasia.^2^, ^3^ Drug‐eluting stents have attempted to prevent neointimal hyperplasia by decreasing VSMC proliferation via inhibition of the cell growth cycle but can have undesirable side effects, including late stent failure, that have limited any widespread clinical use.^1^, ^2^, ^3^ Other approaches or targets are required to address this unmet clinical need.

In neointimal hyperplasia, an increase in growth factors, including PDGF BB (platelet‐derived growth factor BB), results in an increase in proliferative genes and a decrease in smooth muscle marker genes.^3^, ^4^ This change in cell phenotype occurs via ligand engagement of growth factor receptor tyrosine kinases, leading to activation of signaling cascades including the canonical ERK1/2 (extracellular signal‐regulated kinase 1/2) pathway.^4^, ^5^ ERK1/2 is a key regulatory element in the control of transcription following initiation of VSMC proliferation.^4^, ^5^, ^6^ Following its activation, ERK1/2 translocates to the nucleus to directly phosphorylate the Ets transcription factor Elk‐1 (ETS‐like gene‐1), ultimately inducing expression of genes that play primary roles in proliferation.^6^, ^7^, ^8^ Such evidence indicates that growth factor–induced activation of ERK1/2 is critical during the initiation of a proliferative phenotype in differentiated VSMCs. We recently identified an ERK1/2 binding protein, PEA‐15 (phosphoprotein enriched in astrocytes 15, also known as proliferation and apoptosis adapter protein 15), that was previously uncharacterized in the cardiovascular system and that has an important regulatory role in maintaining VSMC phenotype.^9^

Although PEA‐15 has been examined in relatively few cell types to date, studies have revealed that PEA‐15 binds to and sequesters ERK1/2 in the cytoplasm, that is, it acts as a cytoplasmic anchor.^9^, ^10^, ^11^, ^12^ The predominant localization of PEA‐15 in the cell cytoplasm is due to the presence of a nuclear export sequence.^10^ Although PEA‐15 is one of many ERK1/2 binding proteins, it is unique in its anchoring role.^11^, ^12^ PEA‐15 does not alter ERK1/2 activation (as we have shown in VSMCs) but rather its downstream effects, which include nuclear translocation and protein scaffolding.^9^, ^11^, ^12^ PEA‐15 has 2 phosphorylation sites that may be important in altering ERK1/2 binding.^9^, ^13^ In VSMCs, our previous study demonstrated that phosphorylation of these sites by growth factor–induced phospholipase Cγ activation releases ERK1/2 from PEA‐15 binding, allowing ERK1/2 to translocate to the nucleus.^9^ Consequently, this interaction mediates ERK1/2‐induced gene expression.^9^, ^13^, ^14^

In differentiated VSMCs in the absence of growth factors, PEA‐15 prevents ERK1/2 translocation to the nucleus and limits ERK1/2‐mediated gene transcription.^9^ This maintains VSMCs in a quiescent phenotype and could be an important protective mechanism in vivo against the development of vascular disease. In addition, decreased expression of PEA‐15 in VSMCs in culture using small interfering RNA knockdown, results in an increase in proliferation.^9^ In the present study, we hypothesized that PEA‐15 could have important implications for the development of neointimal hyperplasia following angioplasty. To examine this hypothesis, we used a wire injury model in arteries from mice deficient in PEA‐15 *(PEA‐15* ^*−/−*^). The data obtained indicate that PEA‐15 expression is critical for regulating neointimal hyperplasia and may provide a novel therapeutic target to decrease restenosis following angioplasty.

## Materials and Methods

### Transgenic Mice

*PEA‐15* ^*−/−*^ mice were generated by homologous recombination, as described previously.^15^ Animals were maintained on a normal chow diet in specific pathogen‐free conditions. All animal experiments were approved by the University of Aberdeen Ethics Board and performed following UK Home Office license PPL70/8572 under the Animals (Scientific Procedures) Act 1986. Male *PEA‐15* ^*−/−*^ mice were used at 8 weeks of age (n=12 per group), and all experiments were compared with back‐crossed C57BL/6 wild‐type (WT) age‐matched animals (also n=12 per group).

### Cell Culture

Following euthanasia by CO_2_ inhalation and cervical dislocation, aortae were dissected from mice, and primary aortic smooth muscle cultures were established using enzymatic digestion, as described previously.^16^

### Cryosectioning and Confocal Immunomicroscopy

Blood vessels were dissected and incubated (if required) as indicated at 37°C. Tissues were fixed, flash frozen, sectioned, and immunolabeled, as described previously.^16^ All control sections had negligible background staining only. For analysis of immunofluorescent images, 4 fields of view were taken from each sample, and the fluorescence intensity was measured using ImageJ (National Institutes of Health). For routine staining, carotid arteries were fixed overnight in formalin, embedded in paraffin, and cut to 4 μm on a microtome. Slides were stained with hematoxylin and eosin.

### Immunoblots

Arteries were dissected free from connective tissue. Tissue was flash frozen in liquid N_2_ and crushed using a glass homogenizer. Protein extracts were prepared and subjected to SDS‐PAGE followed by immunoblotting, as described previously.^16^

### Electromobility Shift Assay

Denuded aortae were stimulated with PDGF (50 ng/mL) for 30 minutes. Nuclear fractions were prepared, as described previously.^17^ Protein (1 μg) from nuclear preparations was used in each experiment. Protocols were carried out according to the manufacturer's instructions (Active Motif). For competition experiments, cold probe was added in addition to the biotin‐labeled probe. Protein–DNA complexes were resolved on a 6% polyacrylamide gel. Bands were visualized using a streptavidin–horseradish peroxidase detection reagent.

### Carotid Artery Wire Injury

Immediately before surgery, 8‐week‐old male mice received an intraperitoneal injection of an analgesic, buprenorphine (0.1 mg/kg), and 2.5 mg of the antiplatelet drug dipyridamole. Sterile saline (0.5 mL via subcutaneous injection) was also administered to prevent dehydration of the animal during surgery. All surgical procedures were performed under aseptic conditions. Carotid artery injury surgery was performed following an adapted method described by our laboratory.^18^ General anesthesia was induced by 3% (vol/vol) isoflurane supplemented with oxygen (flow rate of 0.5 L/min) and maintained at 1.5% (vol/vol) isoflurane via a face mask throughout the procedure. The depth of anesthesia was monitored throughout the surgery using the hind limb reflex with the other paws secured with tape. A small skin incision was made in the ventral side of the neck to expose the trachea. Blunt dissection was performed to navigate through muscle and connective tissue inferior to the trachea, exposing about 0.5 cm of the left common carotid artery. Precise dissection was used to detach the vagus nerve from the artery, and 2 silk ligatures (size 6‐0) were positioned at the proximal and distal ends of the vessel. The distal ligature was tied tightly, and an arterial clip was positioned at the most proximal end to temporarily occlude the blood flow in the vessel. A small incision was made in the artery, and a piece of a modified flexible nylon wire, adapted by melting the end to create a blunt spherical tip, was inserted into the incision site and held loosely in place by tightening of the proximal ligature. Once the arterial clip was removed, the nylon wire was advanced and rotated down the carotid artery into the thoracic aorta, and this was repeated several times to ensure the removal of the endothelium. Once the nylon wire was removed, the arterial clip was replaced, and the proximal ligature was tied tightly and secured just below the incision site. On removal of the clip, the area was bathed with heparinized saline, and a continuous line of subcutaneous sutures (size 6‐0 [Vircyl; Ethicon]) was used to close the skin incision. For postoperative care, the mice received an intraperitoneal injection of buprenorphine (0.1 mg/kg) and were transferred to a heating mat and maintained at 37°C until recovery. Sham‐operated mice were studied as controls following the same procedure without the insertion of the nylon wire. The contralateral uninjured arteries were also assessed. Mice were euthanized at 7, 14, or 28 days after the carotid artery injury surgery by intraperitoneal injection of 200 mg/kg of sodium pentobarbital to allow for tissue collection.

Neointimal hyperplasia was determined by measuring the intimal and medial layers in arterial sections using ImageJ software.

### Human Saphenous Vein Organ Culture

Saphenous veins were obtained from patients undergoing coronary artery bypass grafting surgery after obtaining written consent under procedures approved by the local ethics committee (National Research Ethic Committee–North of Scotland, reference 06/S0802/26). Veins from 6 different patients were used. These procedures conform to the principles outlined in the Declaration of Helsinki. For ex vivo organ culture, veins were incubated in 30% bovine calf serum and Dulbecco's modified Eagle's medium for 14 days at 37°C in 95% O_2_/5% CO_2_ with medium changed every 48 hours.

### Quantitative Polymerase Chain Reaction

Following treatments, RNA was extracted from human saphenous vein segments cells using TRIzol (Invitrogen), according to the manufacturer's instructions. Quantitative real‐time polymerase chain reaction was performed using TaqMan gene expression assays from Applied Biosystems, as described previously.^9^ Samples were analyzed using a Lightcycler 480 system for real‐time polymerase chain reaction (Roche Applied Biosystems). Changes in gene expression for PEA‐15, smooth muscle myosin heavy chain, and Ki67 were expressed as a ratio of the corresponding GAPDH. The same results were obtained when expressed as a ratio of β‐actin (not shown).

### Materials

Antibodies against ERK1/2, phosphorylated ERK1/2, smooth muscle α‐actin, PEA‐15, proliferating cell nuclear antigen, CD68, Ki67, and GAPDH were from Santa Cruz Biotechnology. All tissue culture reagents were purchased from Life Technologies. All other reagents were purchased from Sigma‐Aldrich.

### Statistical Analyses

Data are expressed as the mean±SEM. Statistical analysis was performed using Prism software (GraphPad Software). For single comparisons, an unpaired *t* test was applied. For multiple comparisons, as appropriate, either 1‐way ANOVA followed by a Newman–Keuls post hoc test or 2‐way ANOVA with a Bonferroni post hoc test was used. *P*<0.05 was considered statistically significant.

## Results

### Phenotype of VSMCs in PEA‐15^−/−^ Mice

The mean arterial pressure, systolic blood pressure, diastolic blood pressure, and heart rate in 8‐week‐old male *PEA‐15* ^*−/−*^ mice were not significantly different from age‐matched WT C57/BL6 male mice. In addition, there was no significant difference in mean heart wet weight (data not shown).

**Figure 1:**
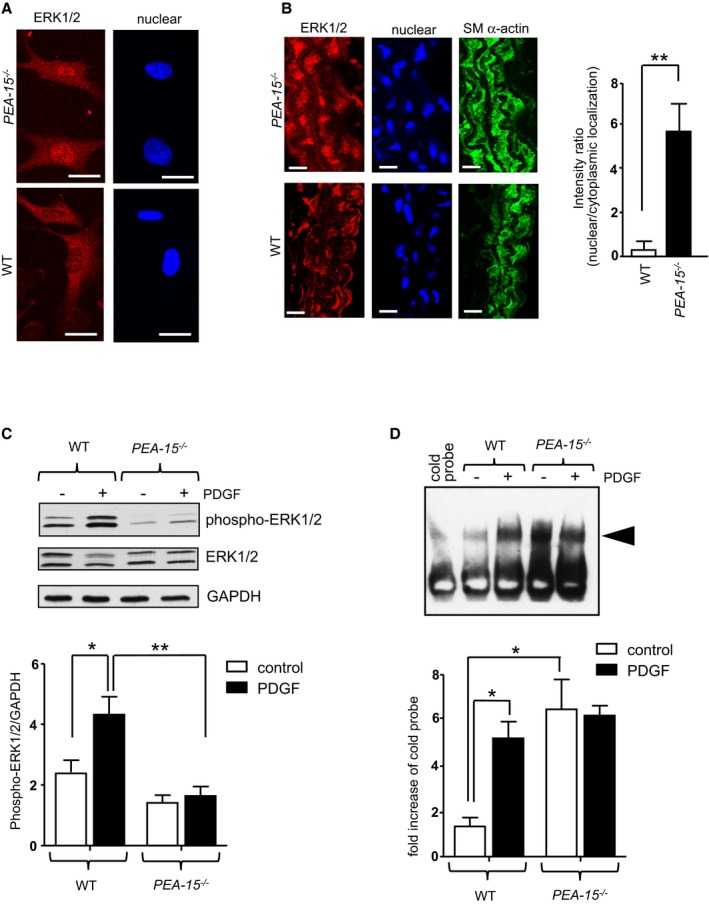
The vasculature from mice deficient in PEA‐15 (phosphoprotein enriched in astrocytes 15; *PEA‐15* ^*−/−*^) has altered ERK1/2 (extracellular signal–regulated kinases 1 and 2) signaling pathways. A, ERK1/2 localization and labeled nuclei in unstimulated aortic smooth muscle cells cultured from *PEA‐15* ^*−/−*^ and wild‐type (WT) mice. Scale bar=5 μm. Representative image from 3 separate cultures. B, Sections of carotid artery from either *PEA‐15* ^*−/−*^ or WT mice, triple‐labeled for ERK1/2, nuclei, and smooth muscle α‐actin. Representative images shown, scale bar=25 μm. Graph displays mean data for nuclear:cytoplasmic ratio calculated using image analyses comparing arteries from 3 mice. **P<0.01 using Student *t* test. C, Activation of ERK1/2 in aortae from *PEA‐15* ^*−/−*^ and WT mice stimulated with 50 ng/mL PDGF (platelet‐derived growth factor) for 15 minutes at 37°C ex vivo. Representative immunoblots for phosphorylated ERK1/2, total ERK1/2, and GAPDH are shown. Mean data of ERK1/2 activation are expressed as a ratio of GAPDH, n=4. *P<0.05 and **P<0.01 using 1‐way ANOVA and Newman–Keuls post hoc test. D, Electromobility shift assay of aortic nuclear extracts from *PEA‐15* ^*−/−*^ and WT aortae to determine Elk‐1 (ETS‐like gene‐1) activation. Aortae were stimulated with 50 ng/mL PDGF for 15 minutes at 37°C ex vivo. The arrowhead on the representative image denotes a gel shift indicating Elk‐1 activation. Mean data are expressed as a fold increase over density of cold probe band (n=3). *P<0.05 using 1‐way ANOVA and Newman–Keuls post hoc test.

We previously showed in cultured human coronary artery VSMCs that a small interfering RNA–induced knockdown of PEA‐15 expression results in increased localization of ERK1/2 to the nucleus and, consequently, an increase in VSMC proliferation.^9^ In this study, ERK1/2 localization in unstimulated VSMCs cultured from the aortae of *PEA‐15* ^*−/−*^ mice was predominantly nuclear (Figure 1A) compared with the cytoplasmic localization in cultured VSMCs from WT mice, in agreement with our previous study. We further examined ERK1/2 localization in freshly isolated arteries from *PEA‐15* ^*−/−*^ mice. Similarly, ERK1/2 was localized predominantly to the nucleus in carotid arteries from *PEA‐15* ^*−/−*^ mice, and this was significantly different from WT arteries with mostly cytoplasmic ERK1/2 distribution (Figure 1B). To determine whether this altered ERK1/2 localization affected ERK1/2 activation, aortae from *PEA‐15* ^*−/−*^ mice were incubated with 50 ng/mL PDGF for 15 minutes, and tissue homogenates were subjected to immunoblotting with anti–phospho‐ERK1/2 antibody (Figure 1C). Interestingly, compared with WT aortae, which produced a significant increase in ERK1/2 phosphorylation following PDGF stimulation, aortae from *PEA‐15* ^*−/−*^ mice did not demonstrate any change in ERK1/2 phosphorylation (although a level of basal ERK1/2 phosphorylation was still maintained). This could be due to ERK1/2 being shielded from upstream activators as a result of its increased nuclear localization. Directly downstream of ERK1/2, the nuclear‐resident transcription factor Elk‐1 is directly activated by ERK1/2 in the nucleus and is important in VSMC proliferation.^6^, ^7^, ^8^ In this study, we sought to determine whether Elk‐1 activation was altered when ERK1/2 was not tethered to PEA‐15 in the cytoplasm. Aortae from *PEA‐15* ^*−/−*^ or WT mice were stimulated with 50 ng/mL PDGF for 15 minutes, and the activation of Elk‐1 transcription was measured using an electromobility shift assay (Figure 1D). In WT aortae, PDGF incubation produced a significant increase in Elk‐1 activation, as expected. However, in aortae from *PEA‐15* ^*−/−*^ mice, Elk‐1 was already significantly activated in unstimulated tissue and did not increase further following PDGF stimulation. This indicates that Elk‐1 activation in VSMCs from *PEA‐15* ^*−/−*^ mice is constitutively increased under resting conditions, presumably as a result of the increased ERK1/2 in the nucleus. The increased ERK1/2 nuclear localization and increased Elk‐1 activation did not result in any changes in arterial structure or wall thickness (data not shown) but could produce altered pathological responses during stress conditions such as vascular injury.

### The Effects of Vascular Injury in PEA‐15^−/−^ Mice

**Figure 2:**
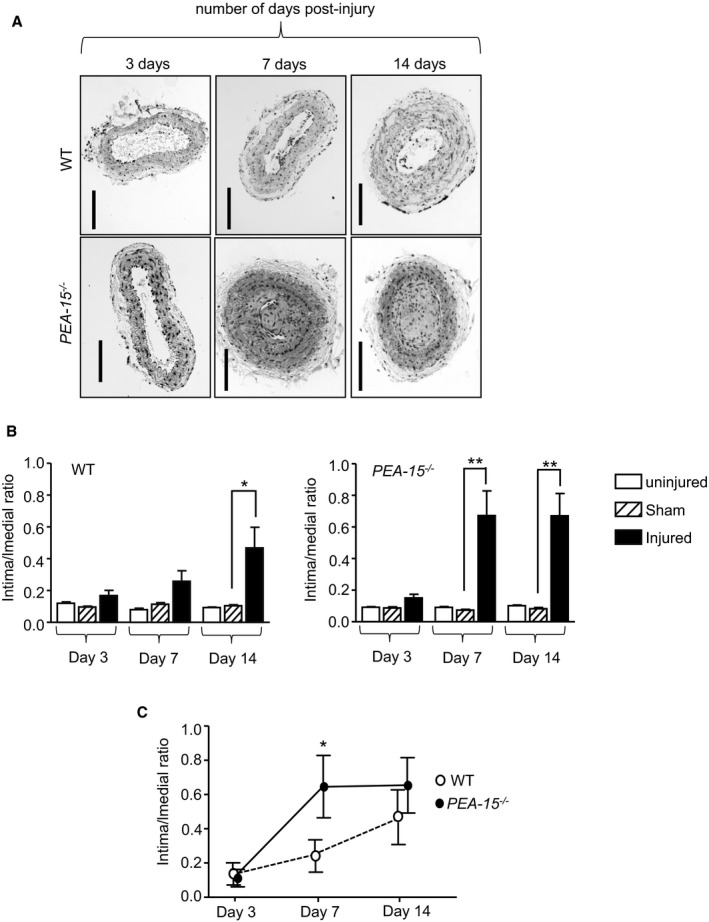
Mice deficient in PEA‐15 (phosphoprotein enriched in astrocytes 15; *PEA‐15* ^*−/−*^) have an increased rate of development of neointimal hyperplasia. A, Representative hematoxylin and eosin–stained sections of mouse carotid arteries from either *PEA‐15* ^*−/−*^ and wild‐type (WT) mice at 3, 7, and 14 days after wire‐induced vascular injury. Scale bar=100 μm. B, Mean data on the intima:media ratio in injured carotid arteries from *PEA‐15* ^*−/−*^ or wild‐type mice at 3, 7, and 14 days following vascular injury (statistical significance is shown only for injured vs sham vessels), n=6. *P<0.05 and **P<0.01 using 2‐way ANOVA and Bonferroni post hoc test. C, Mean data directly comparing the rate of intima development in injured carotid arteries from *PEA‐15* ^*−/−*^ and WT mice, n=6. *P<0.05 using 1‐way ANOVA and Newman–Keuls post hoc test.

*PEA‐15* ^*−/−*^ and WT mice were subjected to wire injury of the left carotid artery for 3, 7, or 14 days. Injured carotid arteries were initially examined using routine microscopy. In the WT mice, the intima:media ratio from injured carotid arteries was significantly different only at 14 days after injury compared with arteries from sham‐operated WT mice or the contralateral uninjured artery but was unchanged at the earlier time points (Figure 2A and 2B). In *PEA‐15* ^*−/−*^ mice, the intima:media ratio was also not different in injured arteries compared with sham/uninjured arteries at 3 days following vascular injury. At both 7 and 14 days after injury, however, there was a significant increase in the intima:media ratio in injured arteries of *PEA‐15* ^*−/−*^ mice compared with either the uninjured contralateral artery or arteries from sham‐operated mice (Figure 2A and 2B). Importantly, when *PEA‐15* ^*−/−*^ and WT mice were directly compared, the rate of neointimal formation was significantly faster, ≈3 times quicker, in *PEA‐15* ^*−/−*^ mice compared with WT mice (Figure 2C). The medial layer thickness and the external elastic lamina were unchanged in *PEA‐15* ^*−/−*^ mice compared with WT mice at all time points examined, indicating that this was predominately caused by increased cellular content (data not shown).

### Expression of Phenotypic Marker Proteins Following Vascular Injury in *PEA‐15* ^*−/−*^ Mice

**Figure 3:**
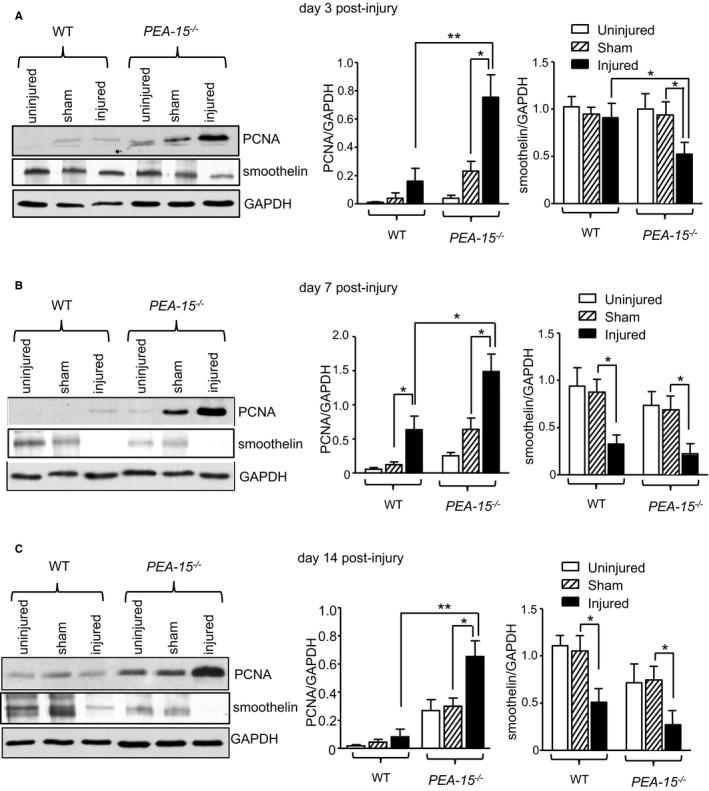
Arteries from mice deficient in PEA‐15 (phosphoprotein enriched in astrocytes 15; *PEA‐15* ^*−/−*^) undergoing neointimal hyperplasia have increased proliferation markers and decreased smooth muscle differentiation markers. The expression of the proliferation marker PCNA (proliferating cell nuclear antigen) and the smooth muscle differentiation marker smoothelin were determined in carotid arteries from *PEA‐15* ^*−/−*^ and wild‐type (WT) mice following vascular injury and compared with the contralateral uninjured artery or arteries from sham‐operated mice. Protein expression was assessed at 3 time points after injury: (A) 3 days, (B) 7 days, and (C) 14 days. Representative immunoblots are shown for each time point, and mean data are expressed as a ratio of GAPDH protein expression, n=3 for each treatment. Statistical comparisons are shown only for sham vs injured arteries. *P<0.05 and **P<0.01 using 2‐way ANOVA and Bonferroni post hoc test for all graphs.

We next examined the expression of proteins relevant to VSMC phenotype: the proliferative marker PCNA (proliferating cell nuclear antigen) and smoothelin, a marker of differentiated smooth muscle cells. The expression of these proteins was examined in carotid arteries from mice following vascular injury at 3, 7, and 14 days, as described, and assessed by immunoblotting of arterial homogenates. PCNA expression was significantly increased and, conversely, smoothelin expression was significantly decreased in injured arteries from *PEA‐15* ^*−/−*^ mice at 3 days after vascular injury compared with sham‐operated and uninjured arteries (Figure 3A). An increase in proliferative markers and a parallel decrease in differentiation markers switch VSMC phenotype towards proliferation, as has been established previously.^4^ In WT mice, however, both PCNA and smoothelin expression was similar in injured arteries compared with sham‐operated and uninjured arteries at 3 days after vascular injury. This early phenotypic switch at day 3 after injury in the knockout animals presumably results in the increased rate of development in the media:intima ratio (compared with WT) observed at day 7 after injury (Figure 2C). At day 7 after injury, PCNA expression was significantly increased in injured arteries compared with sham‐operated or uninjured arteries in both knockout and WT mice; however, increased PCNA expression remained significantly greater in *PEA‐15* ^*−/−*^ mice (Figure 3B). Smoothelin expression was also significantly decreased to the same extent in injured arteries from *PEA‐15* ^*−/−*^ and WT mice after 7 days of vascular injury. Following 14 days of vascular injury, PCNA expression was unchanged in injured arteries from WT mice compared with sham‐operated or uninjured arteries; however, in injured arteries from *PEA‐15* ^*−/−*^ mice, PCNA expression was still significantly increased compared with sham‐operated and uninjured arteries (Figure 3C). Smoothelin expression in injured arteries at 14 days after vascular injury was similarly decreased in both *PEA‐15* ^*−/−*^ and WT mice. These results indicate that, following vascular injury, VSMCs from *PEA‐15* ^*−/−*^ mice switch more rapidly to a proliferative, less differentiated phenotype, and this is sustained throughout neointimal development.

### Cellular Architecture of Neointimal Hyperplasia in *PEA‐15* ^*−/−*^ Mice

**Figure 4:**
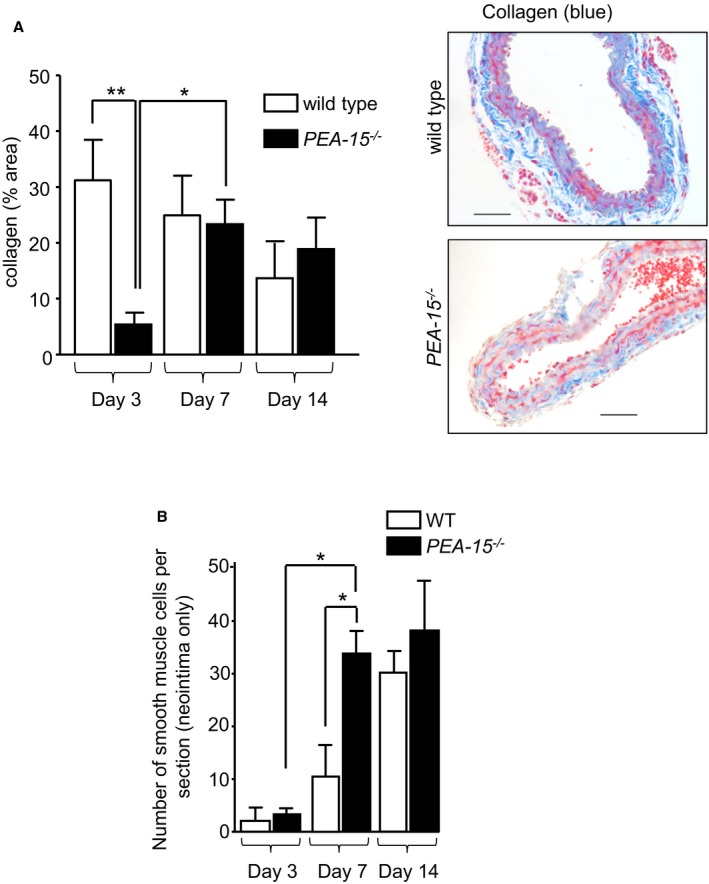
Collagen content is decreased and smooth muscle cell number is increased in arteries from mice deficient in PEA‐15 (phosphoprotein enriched in astrocytes 15; *PEA‐15* ^*−/−*^) undergoing neointimal hyperplasia. A, The collagen content was determined in *PEA‐15* ^*−/−*^ and wild‐type (WT) mice by analyzing sections of arteries stained with Masson's trichrome (representative images are shown). Arteries are compared from mice at 3, 7, and 14 days after injury. Collagen is shown as a percentage of area of the total arterial wall, n=6 for each treatment. *P<0.05 and **P<0.01 using 1‐way ANOVA and Newman–Keuls post hoc test. B, Smooth muscle cell content was assessed by counting the number of cells stained positive for anti–smooth muscle α‐actin antibody in the neointima only (not media) at each time point. This was compared in *PEA‐15* ^*−/−*^ and WT mice at 3, 7, and 14 days after injury, n=4 for each treatment. For clarity, only relevant significant comparisons are shown. *P<0.05 using 1‐way ANOVA and Newman–Keuls post hoc test.

The cellular architecture of injured arteries from *PEA‐15* ^*−/−*^ mice was assessed further by examining the collagen content. Decreased collagen content would allow faster development of neointimal hyperplasia, as migrating cells can move more quickly into neointima. In uninjured arteries, there was no difference in collagen content from WT mice compared with *PEA‐15* ^*−/−*^ mice, as assessed using Masson's trichrome–stained fixed sections (Figure 4A). The collagen content of injured arteries from WT mice did not change over the course of neointimal hyperplasia development; however, in *PEA‐15* ^*−/−*^ mice at day 3 after injury, the collagen content was significantly less compared with WT arteries (Figure 4A). At day 7 following arterial injury, collagen content in *PEA‐15* ^*−/−*^ mice carotid arteries was significantly increased compared with day 3 and was similar to the collagen content in injured arteries from WT mice at day 7 after injury. This was also the case at 14 days after vascular injury. Collagen in sham‐operated arteries was similar to levels in *PEA‐15* ^*−/−*^ and WT mice and unchanged at all time points examined (data not shown).

An increase in the number of smooth muscle cells would indicate that neointimal hyperplasia is, at least in part, caused by VSMC proliferation. The number of smooth muscle cells per arterial cross‐section was determined using sections labeled with anti–smooth muscle α‐actin antibody. Only cells in the neointimal layer were counted. At day 3 following vascular injury, very few smooth muscle α‐actin–positive cells were in the neointima of either *PEA‐15* ^*−/−*^ or WT mice (Figure 4B). At day 7 after injury, the number of smooth muscle α‐actin–positive cells was significantly increased ≈3‐fold in the neointima of arteries from *PEA‐15* ^*−/−*^ mice compared with WT mice. By day 14 after injury, the number of smooth muscle α‐actin–positive cells in the neointima was similar in *PEA‐15* ^*−/−*^ and WT mice. Importantly, in *PEA‐15* ^*−/−*^ mice, there was no significant difference between the number of smooth muscle cells at day 7 compared with day 14; this was maximal at day 7, correlating with the changes in intima:media ratio (as demonstrated in Figure 2). This finding contrasts with a significantly increasing number of smooth muscle cells until day 14 in neointima of WT mice.

### Regulation of PEA‐15 Expression During the Development of Neointimal Hyperplasia in Mice

**Figure 5:**
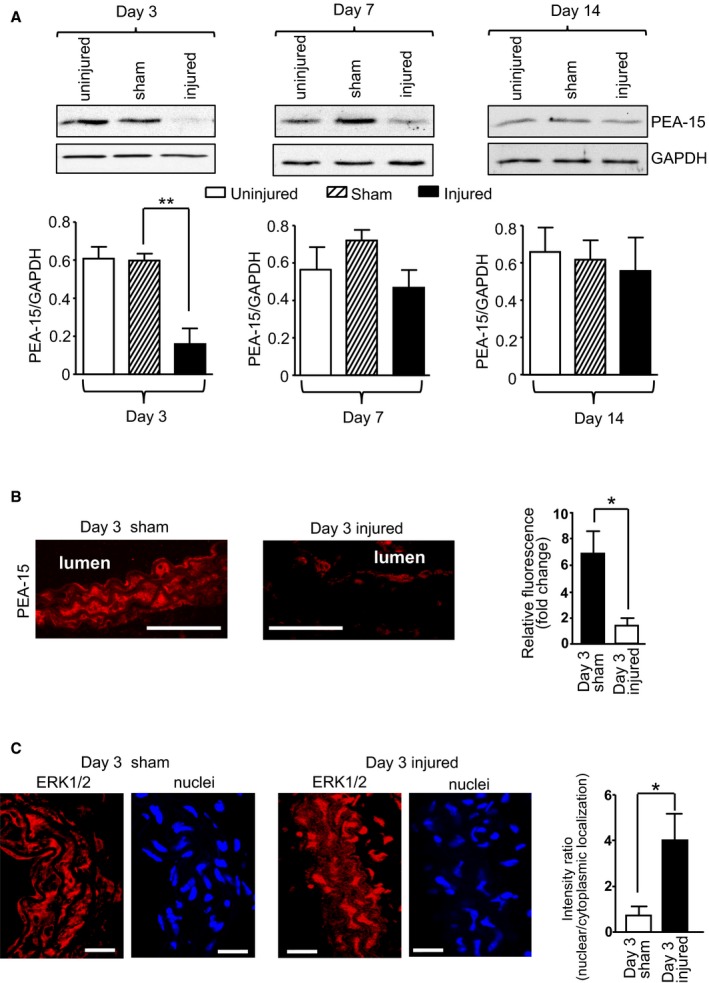
PEA‐15 (phosphoprotein enriched in astrocytes 15) expression is dynamically regulated in mouse arteries during the development of neointimal hyperplasia. A, PEA‐15 expression in injured arteries from wild‐type (WT) mice at 3, 7, and 14 days after injury was compared with expression in arteries from sham‐operated mice and uninjured arteries. Representative immunoblots are shown, and mean data are expressed as a ratio of GAPDH expression (n=6). **P<0.01using 1‐way ANOVA and Newman–Keuls post hoc test. B, PEA‐15 localization and expression was examined in sections from carotid arteries at 3 days following vascular injury in WT mice and compared with arteries from sham‐operated WT mice. Representative confocal images are shown, scale bar=100 mm. Mean data for PEA‐15 expression from confocal images are expressed as fold change in relative fluorescence, n=5. *P<0.05 using Student *t* test. C, ERK1/2 (extracellular signal–regulated kinases 1 and 2) localization in sections of injured arteries from WT mice was compared with sections of arteries from sham animals using confocal immunofluorescence. Representative parallel confocal images are shown for ERK1/2 and DAPI (4′,6‐diamidino‐2‐phenylindole; nuclei), scale bar=25 μm. Mean ERK1/2 localization is presented as the intensity ratio of nuclear/cytoplasmic localization, n=6. *P<0.05 using Student *t* test.

Although *PEA‐15* ^*−/−*^ mice have an increased rate of neointimal development following vascular injury, this would be of pathological relevance only if PEA‐15 expression were regulated during this process in WT tissues. We previously demonstrated that PEA‐15 expression is a critical regulator of VSMC proliferation and differentiation in vitro.^9^ A decrease in PEA‐15 expression in primary cultured VSMCs increases proliferation.^9^ In the current study, we assessed whether the expression of PEA‐15 is altered during the development of neointimal hyperplasia, a process that is dependent on VSMC proliferation.^5^ WT mice were subjected to wire injury of the left carotid artery for 3, 7, or 14 days, as described previously by our laboratory.^18^ The expression of PEA‐15 was examined in injured versus sham‐operated carotid arteries. At 3 days after injury, PEA‐15 expression in carotid arteries was significantly decreased by ≈3‐fold compared with sham‐operated or uninjured arteries, as measured by immunoblotting (Figure 5A). At days 7 and 14 following vascular injury, PEA‐15 expression was not significantly different from that in sham‐operated arteries. This indicates that PEA‐15 expression is decreased at an early time point in neointimal development, before any increase in the intima:media ratio occurs in WT mice (Figure 2B). This early decrease in PEA‐15 expression during neointimal development occurs, at least in part, in VSMCs as assessed by immunolabeling of injured arterial sections with anti–PEA‐15 antibody (Figure 5B). Double‐labeling experiments with anti–smooth muscle α‐actin antibody confirmed these cells in the medial layers as almost exclusively smooth muscle cells (results not shown). This decrease in PEA‐15 expression would be expected to initiate a phenotypic switch in VSMCs toward proliferation, as observed previously in cultured VSMCs.^9^ Based on our data from *PEA‐15* ^*−/−*^ mice (Figure 1), this decreased expression of PEA‐15 should result in increased ERK1/2 localization to the nucleus (Figure 1). We assessed ERK1/2 immunolocalization in arterial sections (Figure 5C). In sections from sham‐operated mice at 3 days, ERK1/2 localization was predominantly cytoplasmic, whereas this switched to predominantly nuclear localization in injured arteries at 3 days (Figure 5C). This was reflected in a significantly increased nuclear:cytoplasmic ratio in injured arteries compared with sham‐operated mice. Such ERK1/2 nuclear localization would increase ERK1/2‐mediated gene transcription, leading to VSMC proliferation (Figure 1).^9^

### Regulation of PEA‐15 Expression in Neointimal Hyperplasia in Human Blood Vessels Ex Vivo

**Figure 6:**
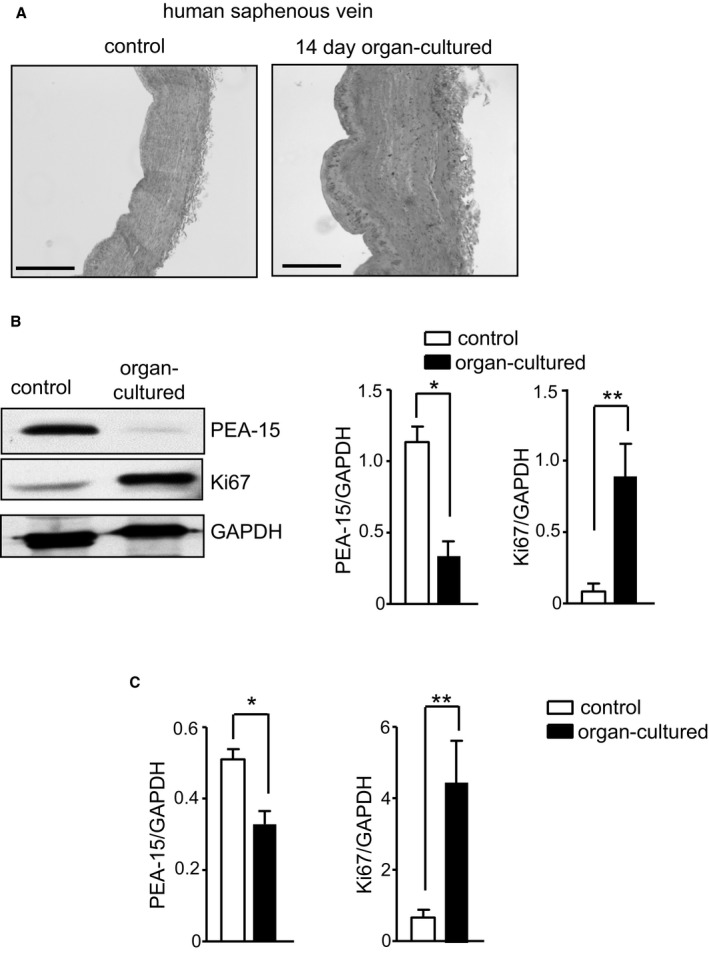
PEA‐15 (phosphoprotein enriched in astrocytes 15) expression is dynamically regulated in human arteries in an ex vivo model of neointimal hyperplasia. A, Representative image of human saphenous vein segments from the same patient, comparing the wall thickness of control and 14‐day organ‐cultured segments. Scale bar=400 μm. B, Immunoblots from control and organ‐cultured vein segments to determine expression of PEA‐15, Ki67 (proliferative marker), and GAPDH. Representative immunoblots are shown. Mean data for PEA‐15 and Ki67 protein expression are calculated as a ratio of GAPDH, n=6. *P<0.05 and **P<0.01 using Student *t* test. C, Gene expression of PEA‐15 and Ki67 in control and organ‐cultured human saphenous vein segments determined by quantatitive polymerase chain reaction. Results are expressed as a ratio of GAPDH, n=6. *P<0.05 and **P<0.01 using Student *t* test.

To determine whether PEA‐15 expression is regulated in neointimal hyperplasia in human blood vessels, we used human saphenous vein incubated ex vivo under conditions that induce hyperplasia, as described previously (Figure 6A).^19^ After 14 days in culture, human saphenous vein sections had decreased expression of PEA‐15, as assessed by immunoblotting (Figure 6B). In addition, a marker of proliferation, Ki67, was significantly increased. We next examined whether the decreased expression of PEA‐15 was a result of *PEA‐15* gene repression. Using real‐time polymerase chain reaction, PEA‐15 gene expression was also significantly decreased (Figure 6C). This was complemented by an increase in *Ki67* gene expression.

## Discussion

The intracellular phosphoprotein PEA‐15 acts as a cytoplasmic tether for ERK1/2, thereby regulating nuclear localization and, subsequently, nuclear‐dependent ERK1/2 signaling.^10^, ^11^, ^12^ We previously demonstrated in VSMCs that this tethering role can potentially regulate phenotypic changes in vitro, leading to an increase in ERK1/2‐dependent transcription and increased proliferation.^9^ In the current study, we demonstrated that PEA‐15 is an important regulator of the response to vascular injury. Mice lacking PEA‐15 develop neointimal hyperplasia at a ≈3‐fold increased rate due to, at least in part, increased proliferation of VSMCs. In addition, in WT mice, vascular injury results in decreased PEA‐15 expression that occurs before significant development of neointimal hyperplasia, indicating a potential role in the initiation of this process. This decreased expression of PEA‐15 was reflected in an ex vivo model of neointimal hyperplasia in human saphenous veins. This indicates that PEA‐15 expression is a vasoprotective mediator in arteries and could limit neointimal hyperplasia at an early stage. In addition, PEA‐15 expression is attenuated following vascular injury, and this decreased expression can drive increased proliferation of VSMCs. This would directly contribute to neointimal hyperplasia and, ultimately, restenosis.

Although *PEA‐15* ^*−/−*^ mice have no obvious cardiovascular phenotype, isolated arteries reveal changes in intracellular signaling that would be expected to potentially regulate vascular structure and function. ERK1/2 localization is significantly shifted toward the nucleus in VSMCs, observed in both primary culture and in freshly isolated arteries. This mirrors our previous findings in cultured human VSMCs, in which PEA‐15 expression was decreased by small interfering RNA, and is similar to those in T‐lymphocytes from *PEA‐15* ^*−/−*^ mice.^9^, ^20^, ^21^ These changes in arteries from *PEA‐15* ^*−/−*^ mice resulted in changes to ERK1/2 activation and signaling by the mitogen PDGF. In VSMCs, although reduced ERK1/2 activation was reflected in a lack of increase in PDGF‐induced Elk‐1 activation, the resting level of Elk‐1 activation was significantly increased in unstimulated arteries from *PEA‐15* ^*−/−*^ mice compared with WT mice. A possible explanation for this apparent dichotomy could be the resting level of phosphorylated (activated) ERK1/2 present in VSMCs. This resting level of activated ERK1/2, coupled with an increased nuclear translocation of total ERK1/2 (some of which will be phosphorylated), could be sufficient to result in this observed increase in basal Elk‐1 activation. Previous studies have shown that elevated basal Elk‐1 would lead to a more proliferative VSMC phenotype.^7^ We found no changes in artery structure in uninjured vessels from *PEA‐15* ^*−/−*^ mice, which presumably reflect adaptation of further downstream mediators allowing normal arterial development. Elevated basal Elk‐1 activation, however, could predispose *PEA‐15* ^*−/−*^ mice to an exaggerated response by vascular stress, namely, the VSMCs persist in a “primed” state. In summary, although no obvious differences were detected in arteries from *PEA‐15* ^*−/−*^ mice, intracellular changes suggest that, under appropriate circumstances, such differences could arise.

In agreement with our proposed role for PEA‐15 in VSMCs, when *PEA‐15* ^*−/−*^ mice were subjected to vascular injury, an increased rate (≈3‐fold) of neointimal hyperplasia was observed. This was maximal at 7 days after injury, compared to a maximal injury response of at least 14 days for WT mice.^18^ The increase in neointimal area was paralleled by an increase in number of smooth muscle cells, indicating that the neointima is predominantly due to an increase in VSMCs. Indeed the majority of cells in the neointima were positive for a smooth muscle marker. Collagen content was decreased in PEA‐15^*−/−*^ mice at this early stage and the consequences of this are not clear. This could affect VSMC migration with lower collagen content presumably enabling faster migration of VSMCs in to the growing neointima.^22^ It is however clear that decreased PEA‐15 expression increases VSMC proliferation in vivo, confirming our previous in vitro study.^9^

Our findings that the accelerated vascular response to injury in *PEA‐15* ^*−/−*^ mice was produced predominantly by proliferation of VSMCs was further supported by a parallel increase in the expression of a proliferative marker and, conversely, a decrease of a VSMC differentiation marker at 3 days after injury. These early changes occurred before any increase in intimal growth was observed. Both changes are known to be directly associated with the initiation of VSMC proliferation.^4^ This suggests that the increased ERK1/2‐dependent transcription (due to its prolonged nuclear localization) in the vasculature of the *PEA‐15* ^*−/−*^ mice is driving VSMC phenotypic change toward a proliferative state at a faster rate. A recent study demonstrated that PEA‐15 downregulation progresses cells through the cell cycle from G1 to S‐phase via increased ERK1/2 nuclear translocation.^23^ ERK1/2 activation is known to be critical in maintaining neointimal formation, as localized ERK1/2 inhibition suppresses the response to vascular injury.^24^ In addition, the reversal of the VSMC proliferative response (as assessed by proliferative marker expression) in WT mice occurs as neointimal development reaches its maximum; however, in the arteries of *PEA‐15* ^*−/−*^ mice, proliferation is maintained throughout the time course (although VSMC proliferation is physically limited because of complete occupation of the lumen). This indicates that PEA‐15 expression could also play an important role in switching off the response to vascular injury. These overall effects of PEA‐15 expression on vascular injury responses generally agree with studies on PEA‐15 in other cell types. In Jurkat T cells lacking PEA‐15 expression, stimulation with interleukin 2 produced an enhanced proliferative response.^20^ In cancer, the role of PEA‐15 is complex and is partially related to phosphorylation status; however, it has been reported that decreased PEA‐15 expression in some cancer cells is related to increased proliferation and invasiveness.^25^, ^26^ Together, the studies cited and our own data indicate that PEA‐15 may be more generally involved in acting as a brake on cell proliferation in responses to several different cellular stresses.

In addition to uncovering the protective role of PEA‐15 in the vasculature, we further revealed that PEA‐15 expression is dynamically regulated during the development of neointimal hyperplasia. Following vascular injury in WT mice, PEA‐15 expression was significantly decreased in VSMCs of carotid arteries at day 3 after injury compared with sham‐operated animals but recovered to levels in sham‐operated animals by day 7. At this early time point, ERK1/2 also had significantly increased nuclear localization that could result in increased VSMC proliferation, as have previously demonstrated in cultured cells.^9^ Because this decrease in PEA‐15 expression occurs before any changes in arterial structure are observed in the injured arteries, a primary role in the vascular injury response is indicated. The delay until day 7 after injury before increased proliferation is physically apparent in the intima:media ratio most likely reflects the time taken from the initiation of the response (including the decreased PEA‐15 response) to sufficient migration and proliferation to follow. The mechanisms involved in the decrease in PEA‐15 expression, possibly via gene repression, are not known. Very few studies have examined the regulation of PEA‐15 expression at the transcriptional level, although hepatocyte nuclear factor 4α has been suggested as a repressive factor.^27^ Further studies are required in VSMCs to fully elucidate PEA‐15 transcriptional regulation. The translational potential of our findings in mice were extended further using an ex vivo model of hyperplasia in human saphenous veins segments. These results demonstrate that PEA‐15 expression is also similarly decreased in human VSMCs under high‐serum conditions, and this may occur, at least in part, via PEA‐15 gene repression. Although this ex vivo model has limitations compared with vascular injury in vivo, it points toward a role in human vascular injury.

Although VSMCs are involved in the accelerated response to vascular injury, other cell types may also have a role including endothelial cells. PEA‐15 is expressed in endothelial cells, and expression may be regulated during phenotypic change, although this is still not clear.^28^ We have not investigated the rate of recovery of the endothelial cell layer following vascular injury; however, increased endothelial cell proliferation (similar to the increased proliferation in VSMCs), may be a benefit and would be expected induce more efficient repair of the endothelial barrier.^2^ Nevertheless, in the current experiments, the overall effect on neointimal hyperplasia in *PEA‐15* ^*−/−*^ mice was detrimental, suggesting that any beneficial effects in other cell types were not sufficient to overcome the increased proliferation of VSMCs. The role of PEA‐15 in other cell types within the vasculature is not clear and requires further investigation.

Taken together our results indicate, for the first time, that PEA‐15 is a protective mediator in the vasculature and acts, at least in part, by inhibiting ERK1/2‐dependent nuclear signaling in VSMCs. This may also occur partly via an inhibitory role in inflammation. In addition, PEA‐15 expression is dynamically repressed in the early stages of neointima hyperplasia, indicating a primary role in this injury process. This evidence indicates that PEA‐15 is a novel therapeutic target to prevent restenosis. Maintaining PEA‐15 expression immediately following angioplasty and stent placement could protect the vasculature at the critical initiation stage of neointimal hyperplasia when the physiologically protective role of PEA‐15 is lost.

## Sources of Funding

This work was supported by a grant from the Medical Research Council, UK (MR/K012789/1).

## Disclosures

None.
